# Commentary: Coding of serial order in verbal, visual and spatial working memory

**DOI:** 10.3389/fpsyg.2018.02330

**Published:** 2018-11-21

**Authors:** Elger Abrahamse, Alessandro Guida

**Affiliations:** ^1^Basque Center on Cognition, Brain and Language, San Sebastián, Spain; ^2^IKERBASQUE, Basque Foundation for Science, Bilbao, Spain; ^3^LP3C, Université Rennes, Rennes, France

**Keywords:** working memory, serial order, spatial coding, position markers, mental whiteboard hypothesis

The ordinal position effect refers to the observation that the later an item is positioned within a sequence maintained in working memory (WM), the larger is the rightward bias that it generates upon later retrieval during diagnostic tasks intended to capture this bias (van Dijck and Fias, 2011; van Dijck et al., 2013; Guida et al., 2016, 2018b). Recently, Ginsburg et al. (2017) showed how this effect replicates: for verbal but not spatial sequences (Experiments 1–3); for both abstract and concrete stimuli whenever they are verbalized (Experiment 4); for conditions with and without articulatory suppression (Experiment 5); and finally for pseudo-words whenever they are semantically encoded (Experiment 6). Since lack of semantic processing is a common denominator across their conditions with absent ordinal position effects, Ginsburg et al. concluded that item sequences are spatially maintained in WM only “if the items memorized in WM are semantically coded by the participants” (p. 647). In this commentary we outline our concern with this conclusion, and specifically with the interpretation of absent ordinal position effects.

The ordinal position effect is interesting for two reasons. First, retrieval generates spatial codes *spontaneously* in the absence of explicit spatial cues or instructions. Accordingly, the Mental Whiteboard Hypothesis (MWH) was proposed: Serial order demands probe an internally generated spatial template—the metaphorical “mental whiteboard”—onto the coordinates of which items can be systematically mapped (Abrahamse et al., 2014, 2017). Encoding, maintenance, refreshing, and selection processes in serial order WM, then, depend on internal spatial attention. Second, spatial coding for serial order is sufficiently systematic between (Western) subjects to produce on average a left-to-right orientation–despite alternatives such as right-to-left (Guida et al., 2018b; Guida et al., in revision) or vertical orientations (Dutta and Nairne, 1993; Zhou et al., 2018; see also Darling et al., 2017), and despite potential individual differences in orientations (Cooperrider et al., 2017). We believe that the “classic” (left-to-right) ordinal position effect reflects a default orientation in Western subjects that is induced by (a weighted sum of) shared ontological experiences such as reading direction (Guida et al., 2018b), keyboard use (Darling et al., 2017), or spatial metaphor use (Zhou et al., 2018). Yet, this default may be flexibly overwritten, for example by task-specific directional cues such as right-to-left presentation of items on the screen (Guida et al., in revision).

Whereas, the default orientation allowed for the discovery of the ordinal position effect, the absence of left-to-right coding for serial order is not necessarily informative in itself: Does this mean there is no spatial coding overall, or is spatial coding oriented differently—and/or with larger individual differences—such that horizontally outlined response options cannot capture spatial coding? This consideration suspends the conclusion by Ginsburg et al. (2017) that spatial coding for serial order requires semantic processing. Let us consider some of their findings.

Experiments 1–3 showed no left-to-right *re*coding for sequences of 4 black dots displayed on an (invisible) 8 × 8 matrix. Does this indicate that participants did not use spatial coding? Not necessarily, as recoding may not be necessary: The mental whiteboard can be flexibly used across the horizontal and vertical axes to encode the dot-location sequence based on locations at which dots were physically presented, and with pathway information being used to capture order information. Spatial cognition being reused for (non-spatial) serial order does *not* imply that the former starts to be dictated by the latter.

Experiment 4 showed a classic ordinal position effect for verbalized abstract drawings, but not for non-verbalized ones. Yet, nothing refutes the possibility of different spatial coding in the non-verbalized items. Indeed, instead of reflecting a critical role for semantic processing *per se*, these findings may relate to an impact of reading direction in shaping the use of the mental whiteboard (Guida et al., 2018b). Hence, verbalization triggers the language system, and as such generates systematic left-to-right spatial coding in Western participants due to shared reading experience. Without such verbalization-induced left-to-right systematicity across subjects, spatial coding may occur with different orientations (e.g., vertical coding) and/or with larger individual differences.

Experiment 6 showed the ordinal position effect for pseudo-words, but only when semantically encoded. Here, the absent effect for non-semantically encoded pseudo-words may relate to chunking rather than a lack of sematic processing. The (left-to-right) ordinal position effect is constrained by sequence length (Huber et al., 2016; Guida et al., 2018a), and effective length of sequences of pseudo-word may differ according to the extent that semantic encoding allows for efficient chunking strategies. Hence, for serial order maintenance of non-chunked pseudo-words, the use of the mental whiteboard might just differ–even when the verbal nature of items probes left-to-right coding (see above). Indeed, Ginsburg et al. (2017) report an almost significant interaction between serial order and spatial processing for not-semantically-related pseudo-words, with positions 2 and 3 in the sequence responded to faster with the right hand but no left-right difference for positions 1 and 4. This hints at spatial coding that just does not follow the classic left-to-right orientation.

Overall, we believe that Ginsburg et al. (2017) prematurely concluded that spatial coding is only used for serial order WM when items of the sequence are semantically processed. Yet, their study has clear value. First, as both the phonological loop and attentional refreshing have been proposed as mechanisms of maintenance in verbal working memory, the ordinal position effect obtained under conditions of articulatory suppression (i.e., Experiment 5) suggests that spatial coding of serial order in verbal working memory is related mainly to the latter of these two maintenance mechanisms (Abrahamse et al., 2017). Second and more importantly, the study reveals the need for future studies to consider both a broader coverage of space including the vertical axis (e.g., via eye-tracking; Rinaldi et al., 2015), and coding in individually idiosyncratic manner. Only then will the reliable absence of the ordinal position effect become theoretically valuable.

## Author Contributions

All authors listed have made a substantial, direct and intellectual contribution to the work, and approved it for publication.

### Conflict of Interest Statement

The authors declare that the research was conducted in the absence of any commercial or financial relationships that could be construed as a potential conflict of interest.
